# The rib index practically is not affected by the distance between the radiation source and the examined child

**DOI:** 10.1186/1748-7161-10-S1-O44

**Published:** 2015-01-19

**Authors:** Theodoros B Grivas, Konstandinos Soultanis, Christina Mazioti, Vasileios Kechagias, Antonios Akriotis, Konstandinos Athanasopoulos, Christos Naskas

**Affiliations:** 1 Department of Orthopedics and Traumatology Tzaneio General Hospital, Greece; 2 Department of Orthopaedics University of Athens University General Hospital “Attikon”, Greece

## Introduction

All lateral spinal radiographs in idiopathic scoliosis (IS) show a Double Rib Contour Sign (DRCS) of the thoracic cage, a radiographic expression of the rib hump. The outline of the convex overlies the contour of the concave ribs. The rib index (RI) method was extracted from the DRCS to evaluate rib hump deformity in IS patients. The RI was calculated by the ratio of spine distances d1/d2 where d1 is the distance between the most extended point of the most extending rib contour and the posterior margin of the corresponding vertebra on the lateral scoliosis films, while d2 is the distance from the least projection rib contour and the posterior margin of the same vertebra, (Grivas et al 2002). In a symmetric thorax the “rib index” is 1.

## Aim

This report is the validity study of DRCS, ie how the rib index is affected by the distance between the radiation source and the irradiated child.

## Methods

The American College of Radiology's (2009) guidelines for obtaining radiographs for scoliosis in children recommends that the scoliotic - films distance to be 1,80 meters.

As normal values for the transverse diameter of the ribcage in children aged 6-12 years were used those reported by Grivas in 1988.

## Results

**Figure 1:**
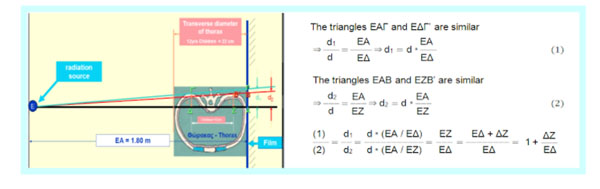


Using the Euclidean geometry (Figure 1) it is shown that in a normal child 12 years of age, provided that the distance ΔZ ≈ 12cm (11,84) and EA = 180cm, with transverse ribcage diameter of the child 22 cm, then d1/d2 = 1.073.

## Conclusions

This validity study demonstrates that the DRCS is substantially true and practically the RI is not affected by the distance between the radiation source and the irradiated child. The RI is valid and may be used to evaluate the effect of surgical or conservative treatment on the rib cage deformity (hump) in children with IS. It is noted that IR is a simple method and a safe reproducible way to assess the rib hump deformity based on lateral radiographs, without the need for any other special radiographs and exposure to additional radiation.

## Disclosure

No conflict of interest
